# Friction and Wear Performance of Staple Carbon Fabric-Reinforced Composites: Effects of Surface Topography

**DOI:** 10.3390/polym12010141

**Published:** 2020-01-06

**Authors:** Chang-Mou Wu, Yi-Ching Cheng, Wen-You Lai, Po-Hsun Chen, Tzong-Der Way

**Affiliations:** 1 Department of Materials Science and Engineering, National Taiwan University of Science and Technology, Taipei 10607, Taiwan; cmwu@mail.ntust.edu.tw (C.-M.W.); chris01_lay@hotmail.com.tw (W.-Y.L.); jason616568@gmail.com (P.-H.C.); 2 Research and Development Center for Smart Textile Technology, National Taipei University of Technology, Taipei 10608, Taiwan; 3 Department of Biological Science and Technology, College of Biopharmaceutical and Food Sciences, China Medical University, Taichung 40402, Taiwan; tdway@mail.cmu.edu.tw

**Keywords:** staple carbon fiber fabric, hybrid composites, impregnation ratio, surface topography, friction and wear

## Abstract

Here, staple carbon fiber fabric-reinforced polycarbonate (PC)- and epoxy (EP)-based composites with different impregnating resin levels were fabricated using a modified film stacking process. The effects of surface topographies and resin types on the tribological properties of stable carbon fabric composites (sCFC) were investigated. Friction and wear tests on the carbon composites were conducted under unlubricated sliding using a disk-on-disk wear test machine. Experimental results showed that the coefficient of friction (COF) of the sCFC was dominated by matrix type, followed by peak material portion (S_mr1_) values, and finalized with core height (S_k_) values. The COF of composites decreased by increasing the sliding speed and applied pressure. This also relied on surface topography and temperature generated at the worn surface. However, the specific wear rate was strongly affected by resin impregnation. Partially-impregnated composites showed lower specific wear rate, whereas fully-impregnated composites showed a higher wear rate. This substantially increased by increasing the sliding speed and applied pressure. Scanning electron microscopy observations of the worn surfaces revealed that the primary wear mechanisms were abrasion, adhesion, and fatigue for PC-based composites. For EP-based composites, this was primarily abrasion and fatigue. Results proved that partially-impregnated composites exhibited better tribological properties under severe conditions.

## 1. Introduction

Friction and wear performance play a key role in tribo-component applications. High-quality friction materials must possess properties such as stable coefficient of friction (COF), low wear rate, rapid heat dissipation, and low cost [1,2]. To satisfy these parameters, reinforcing fibers are commonly used in friction washers to improve tribological and mechanical properties such as thermal stability, strength, and stiffness [3,4,5]. Different fibers such as cellulose [6], Kevlar [7], ceramic [8], carbon [9,10,11], and glass [12,13,14], have also been incorporated into friction materials, leading to improvements in tribological properties. Carbon fibers specifically are used as reinforcement due to their distinctive properties such as self-lubrication, chemical inertness, high strength and modulus, and excellent thermal stability [10,15]. Many papers have reported on the tribological performance of polymer–matrix composites with short or continuous carbon fibers [16,17,18,19]. The friction behaviors of the staple carbon fiber composites have been reported having stable behavior [20]. Moreover, oxidized polyacrylonitrile fibers were used to produce staple carbon fibers by employing a continuous negative pressure carbonization method to the weaved fabrics in order to prepare carbon fiber fabrics. Compared to the conventional multifilament version, staple carbon fabric is advantageous for its low production costs. However, to the best of the authors’ knowledge, the number of reports studying the influences of the staple carbon fiber fabric formed by low-temperature carbonization on the friction and wear properties is scarce.

Polymer materials have an excellent strength-to-weight ratio and possess excellent tribological properties. Examples include thermoplastics such as polybutylene terephthalate (PBT) [17], polyamide (PA) [12,21], polyetheretherketone (PEEK) [22,23], and polycarbonate (PC) [24,25]. Most of these materials are semi-crystalline polymers that have a low glass transition temperature (Tg). As an amorphous polymer, PC is a widely used thermoplastic due to its high Tg, hardness, and toughness. However, there have been few studies on the friction and wear behavior of PC as the matrix of composite material. Additionally, thermosets such as epoxy (EP) [26,27,28] and phenolic [4,29] have also been the subject of many studies. EP is widely used since it possesses high mechanical strength and hardness, and good chemical stability. Therefore, PC and EP were chosen as the matrix of composite material for comparing tribological properties in this study.

The effect of surface topography on tribological performance is significant. Most literatures discuss the tribological performance of surface glossy of fully-impregnated composites with short or continuous carbon fiber reinforcement [5,26]. Some works studied the effect of porous surface topography on the tribological performance of carbon fiber composites [18,19,30]. The influence of the correlation between surface topography of the asperity height distribution and friction/wear was also investigated [15,31,32]. Few papers have reported the effect of carbon fiber-dominated surface topography on the tribological performance of composites. In this study, the partially-impregnated level was used to prepare carbon fiber composites. Their surface topography and tribological performance were evaluated and compared with fully-impregnated composites.

For tribologically loaded components, properties like the COF, specific wear rate, surface topography, and heat dissipation of the carbon fiber composites drive their acceptability for industrial applications. To determine the effect of resin matrices and surface topography on tribological performance, four kinds of stable carbon fabric composites (sCFC) were investigated under the unlubricated sliding condition with a disk-on-disk test method. Finally, thermal properties, surface topographies, and wear mechanism of composites were also characterized.

## 2. Materials and Methods

### 2.1. Materials

**Figure 1:**
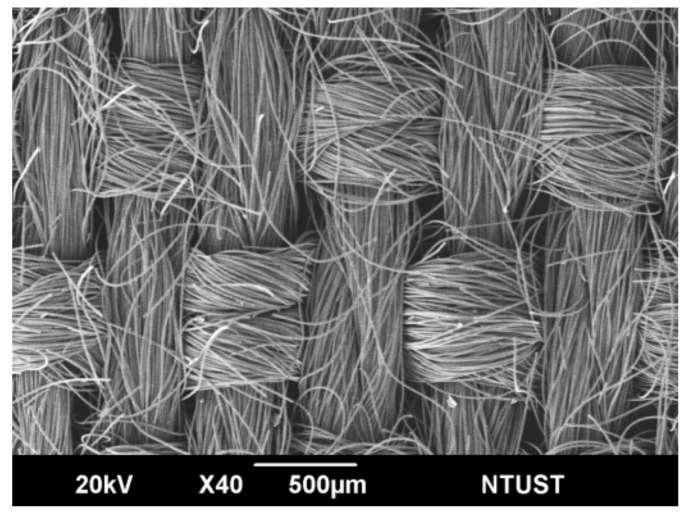
SEM micrographs of short carbon fiber fabric.

Staple carbon yarns were manufactured with polyacrylonitrile oxidized fiber staple yarns, using the direct spinning method. This was done by going directly from tow to spun yarn in a stretch-breaking, drafting, and twisting operation. The 12K oxidized filaments were stretch broken through a stretch-breaking process using a pacific conversion system to form staple fibers tow. These tows were then woven into plain fabric, as shown in Figure 1. The short carbon fabric had an areal weight of 160 g/m^2^, and an 85% carbonization degree. The densities of the warp and weft were 14.2 ends/cm and 11.0 picks/cm, respectively. To support the material, glass fiber fabrics with an areal weight of 81 g/m^2^ were used. Commercially available resins PC (thermoplastic) and EP (thermoset) were used as the matrix. With an areal weight of 40 g/m^2^, EP was a one-component resin for hot melt processing and was casted into the film. The glass transition temperature, Tg, of EP resin was approximately 125 °C. A commercial-grade thermoplastic PC film, with an areal weight of 210 g/m^2^, was selected. The glass transition temperature of PC resin was 150 °C.

### 2.2. Sample Preparation

**Figure 2:**
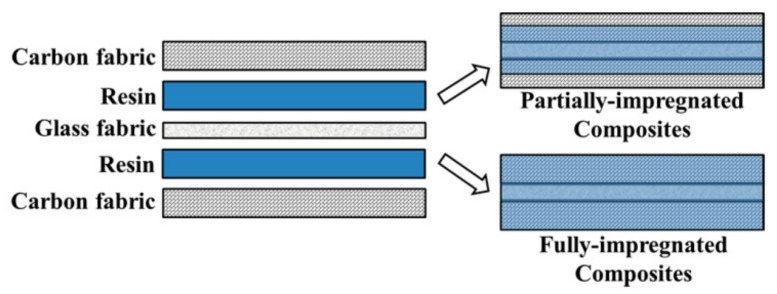
Structure of hybrid composites.

The sCFC were interply hybridized by film stacking using two layers of sCF sandwiched with one layer of glass fabric, and bonded by either the PC or EP matrix (Figure 2). PC composites were prepared by hot pressing at 230 °C for 1 min under a pressure of 10 MPa. EP composites were prepared by hot pressing at 150 °C for 30 min under a pressure of 5 MPa. The composites were then cooled in a compressed condition and then cut with a cutter for the thermal and tribological tests. The composites with different impregnated levels were controlled by resin film thickness or areal weight. The total PC film thickness was 0.6 mm for fully-impregnated (PC-F) and 0.25 mm for partially-impregnated (PC-P) composites samples. The areal weight of the EP film was 0.8 g/m^2^ for fully-impregnated (EP-F) and 0.6 g/m^2^ for partially-impregnated (EP-P) composites samples. The composition frictions (vol%) of sCFC used in this study are listed in Table 1. The porosity of the samples was determined by liquid permeation. An average of five readings was taken for each sample.

### 2.3. Characterization of the Composites

#### 2.3.1. Microstructure Test

The three-dimensional topographies of the composites with different roughness were observed under the confocal laser scanning microscope (CLSM, OLS5000, Olympus, Tokyo, Japan). The surface roughness (S_q_) is the root mean square height of the surface and was measured in 2400 × 2400 µm^2^. Surface functional parameters such as core height (S_k_), reduced peak height (S_pk_), and peak material portion (S_mr1_) were also measured by CLSM, and are all derived from a bearing ratio curve based on the ISO 25178-2 standard [33]. The bearing ratio is the percentage of the total evaluation length of surface profile corresponding to regions where profile height exceeds a given value showing the Abbott–Firestone curve of a surface with a Gaussian height distribution. S_k_ represents the core height where the load will be primarily distributed. S_pk_ represents the surface material that may be worn out through initial contact with the counterpart surface. S_mr1_ is obtained by tracing a horizontal segment from the intersection of the line of the minimum slope with the vertical axis to the bearing ratio curve and represents the percentage of the initial contact surface.

A scanning electron microscope (SEM, JSM-6390LV, JEOL, Tokyo, Japan) was used to observe the topographies of worn surfaces. Prior to SEM observations, samples were mounted on aluminum stubs and sputter coated with a thin layer of gold to prevent electrical charging. SEM micrographs were taken at various magnifications using a 20 kV acceleration voltage.

#### 2.3.2. Thermal Properties Test

Thermal properties of composites were obtained using an Alambeta instrument (Sensora, Liberec, Czech Republic). Tests performed according to the standard ASTM D7984. An average of five readings were taken for each sample. The thermal properties of composites are calculated by the equations provided by Frydrych, Dziworska, and Bilska [34].

#### 2.3.3. Tribological Properties Test

**Figure 3:**
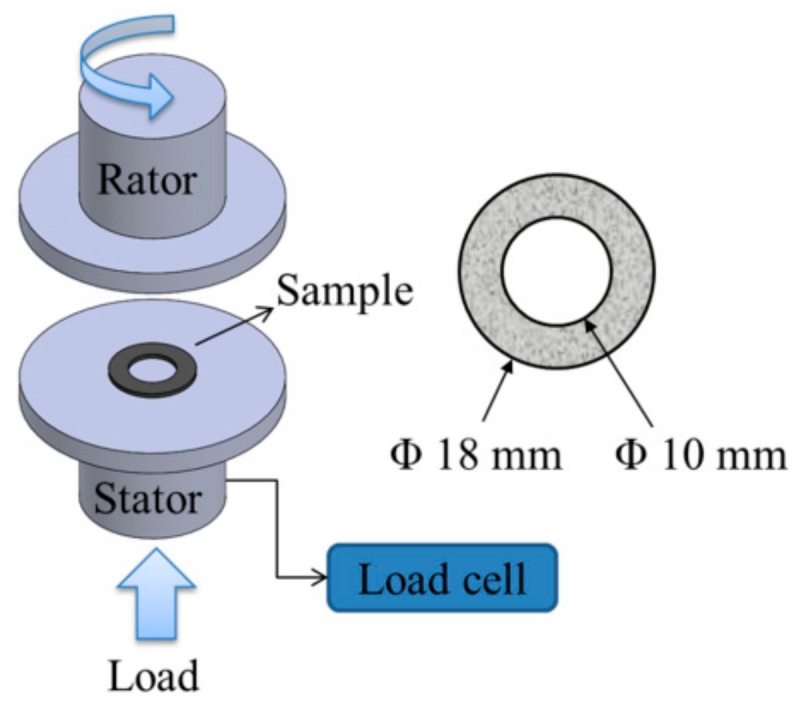
Schematics of wear tester and sample specification.

The tribological characteristics of the composites were investigated under dry wear sliding conditions using an in-house disk-on-disk sliding wear test machine (Figure 3). Friction and wear tests were conducted at various impregnation structures (partially- and fully-impregnated), applied pressures (0.5, 1.0, and 1.5 MPa), sliding speeds (500, 1000, and 1500 rpm), and constant engagement cycles (10,000 cycles). The specimens had an outer diameter of 18 mm and an inner diameter of 10 mm. One unit rpm is equivalent to an average linear speed of 0.044 m/min. The steel counterpart is made of AISI SAE 4140 alloy steel (a chromium molybdenum alloy steel (carbon content: 0.38%–0.43%) with a 55 mm diameter). The surface roughness of the root mean square deviation (S_q_) is 0.33 μm. Five tests were conducted for each sample.

To minimize data scattering, the wear of 10,000 cycles was selected for all samples. An experimental data average between 2000 and 10,000 cycles was used to determine the dynamic friction coefficient and was calculated by the following equation:

(1) $$\mu_{d}=\frac{3M_{d}}{2\pi p\left(R_{0}{}^{3}-R_{i}{}^{3}\right)}$$

where *μ_d_* is the dynamic friction coefficient, *M_d_* is the dynamic friction torque (Nm), *p* is the engagement pressure (MPa), *R*_0_ is the outside radius (mm), and *R_i_* is the inner radius (mm).

The specific wear rate *K_s_* (cm^3^/Nm) was determined using the following relation:

(2) $$K_{s}=\frac{△m}{\rho FL}$$

where ∆*m* is the weight loss (g), *ρ* is the density (g/cm^3^), *F* is the average friction force (N), and *L* is the sliding distance (m).

## 3. Results and Discussion

### 3.1. Influence Mechanism of Bearing Ratio Curve and Surface Topography on Friction and Wear

**Figure 4:**
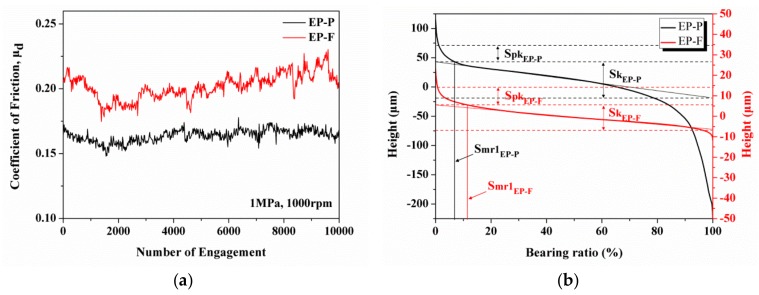
The (**a**) friction coefficient curves and (**b**) bearing ratio curves of partially-impregnated (EP-P) and fully-impregnated (EP-F) epoxy (EP)-based composite samples.

The surface roughness parameter values for PC- and EP-based sCFC are shown in Table 2. The surface roughness, S_q_, of PC-F and of EP-F (4.39 and 4.23 μm) is significantly lower than of PC-P and of EP-P (23.95 and 51.25 μm). The partially-impregnated composites where the surface topography is carbon fiber-dominated have higher S_k_ and S_pk_ values than the fully-impregnated composites. This indicates a broad carbon fiber height distribution, especially for EP-P (69.40 and 32.43 μm). If the surface topography of carbon fabric composites lacks load-carrying capability, the S_pk_ might be worn out in the initial contact with the counterpart surface. The COF curves and bearing ratio curves of EP-P and of EP-F were chosen for comparison, as shown in Figure 4. The bearing ratio curve of EP-P is steeper than EP-F, which indicates a larger average height difference. Despite the higher S_k_ values of EP-P, its COF is still lower than EP-F (Figure 4a). This indicates that S_k_ values are not the primary influence factor for COF. The S_mr1_ value of EP-F is 13.25%, which is higher than the value of EP-P (7.36%). This indicates that EP-F has a higher percentage of wearing contact area, which, in turn, resulted in a higher COF (Figure 4b). The COF shows a positive correlation with the bearing area ratio which was also reported by Zhu et al. [35]. These results prove that S_mr1_ influences the COF and these values should be taken into consideration when developing a friction composite. Furthermore, the surface topographies of fully-impregnated composites were smooth, thus the surface roughness being almost similar. Although the friction behavior was affected by surface topographies, the resin type still dominates the friction properties.

### 3.2. Effect of Sliding Speed on Friction Coefficient and Specific Wear Rate

The COF and specific wear rate of sCFC as a function of sliding speed under the applied pressure of 1 MPa were summarized in Table 3. The COFs of PC-based composites are higher than those of EP-based composites, indicating that resin type is the primary COF influencing factor. This may be attributed to surface behavior and the intrinsic property of PC resin where it becomes highly viscous at low applied pressures and sliding speed. The S_mr1_ values are the secondary COF influence factors. As shown in Table 2 and Table 3, a lower S_mr1_ has a lower COF, especially for EP-P. When the S_mr1_ values are close, the COF was dominated by S_k_ values. A higher S_k_ represents a higher COF.

**Figure 5:**
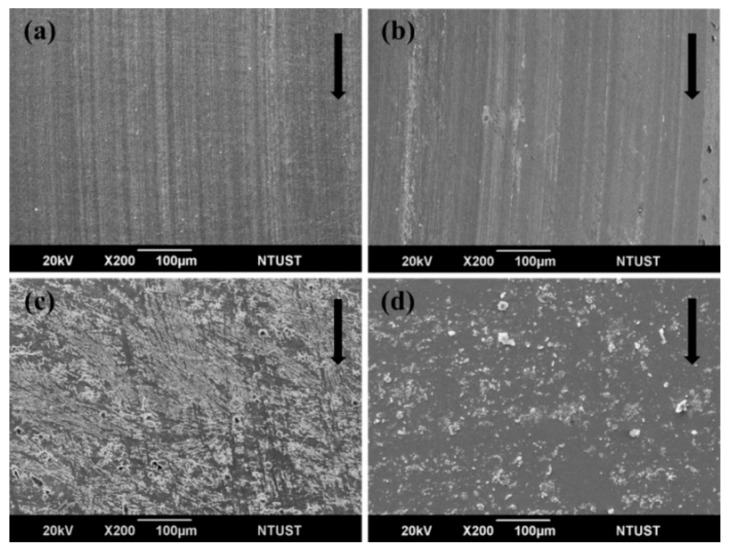
SEM images of the transfer phenomenon on the counterpart surface of (**a**) partially-impregnated PC-based composite samples (PC-P), (**b**) EP-P, (**c**) fully-impregnated PC-based composite samples (PC-F), and (**d**) EP-F under a pressure of 1 MPa and sliding speed of 1500 rpm. The black arrows indicate the sliding direction.

Furthermore, the COF of sCFC decreased with increasing sliding speed. For the PC-P, a similar COF under the sliding speeds of 500 and 1000 rpm was observed. The COF decreased from 0.27 to 0.21 as sliding speed increased to 1500 rpm. This was attributed to frictional heating under severe conditions, which leads to a thermal softening of the polymer. Meanwhile, a strip-like transfer layer on the steel counterpart surface was observed, composed of the carbon content shown in Figure 5a. The specific wear rate of PC-P was very low and did not increase with increasing sliding speed, but remained in the range from 8.4 × 10^−7^ to 1.5 × 10^−6^ cm^3^/Nm (Table 3). The surface topography of PC-P was constructed with bare carbon fibers and formed a high porosity (39 vol%) structure. The thermal diffusivity of PC-P is 0.053 mm^2^/s. The frictional heat during the friction process can quickly dissipate through the high heat conductivity of carbon fiber and air convection avoiding mechanical or fiber damage caused by excessive heat accumulation.

**Figure 6:**
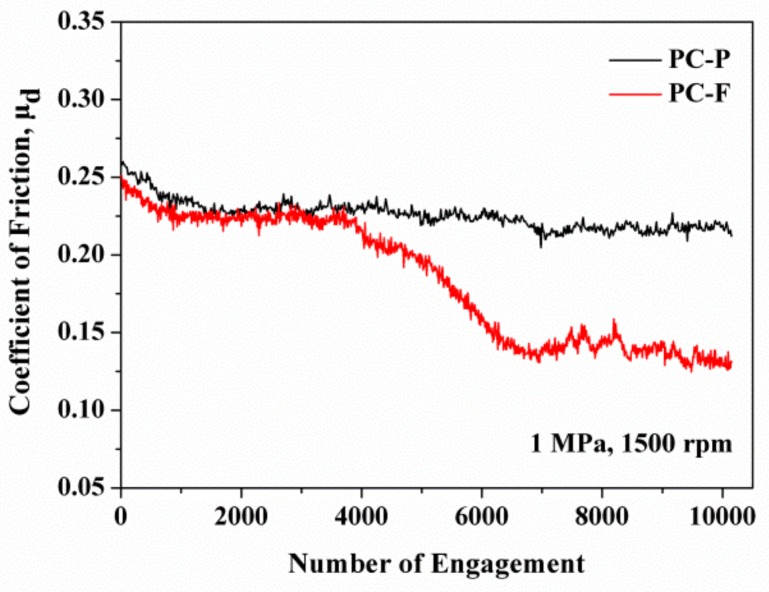
Influence of transfer film on friction coefficient of PC-P and of PC-F at an applied pressure of 1 MPa and sliding speed of 1500 rpm.

For the PC-F, the COF decreased to 11%, from 0.26 to 0.23, with a sliding speed of 1000 rpm. Under the severe sliding speed of 1500 rpm, the COF decreased to 39% from 0.23 to 0.14. These results show that COF is highly influenced by the thermal softening of the PC matrix. As the sliding speed increases, the frictional heating builds up rapidly and causes thermal softening forming of the PC transfer film. This can result in lower shear strength, reducing the friction force resisting the sliding of the counterpart. The friction-induced temperature rise may also accelerate the transfer of PC resin to the counterpart surface. The wear debris of PC-F presents a large patchy-like non-uniform transfer film on the steel counterpart surface (Figure 5c). As shown in Figure 6, the COF of PC-P maintains a steady-state during the whole wearing process, but the COF of PC-F exhibits a two-stage transformation phenomenon, while the COF drops from 0.24 to 0.14 after 6000 cycles. The formation of resin transfer films on the steel counterpart has been reported as a positive mechanism for the reduction of COF in the case of polymer-based composites [17]. The specific wear rate of PC-F significantly increased from 9.3 × 10^−6^ to 2.1 × 10^−5^ cm^3^/Nm, in comparison to PC-P, by increasing the sliding speed. This indicated that, while the specific wear rate of sCFC is sensitive to resin impregnation, the COF is not. Therefore, resin impregnation is the primary influence factor for the specific wear rate. As shown in Table 4, PC-F exhibited the highest thermal absorptivity (404.7 Ws^1/2^/m^2^K). These results indicated that the higher thermal absorptivity of PC-F causes thermal accumulation, and consequent softening of the PC resin, leading to a large amount of wear debris and to carbon fabric composites failure.

EP-P showed the lowest COF (approximately 0.24), which was attributed to also having the lowest S_mr1_ (7.36%). This led to fewer carbon fibers that participated in the wear behavior, thus reducing COF. As the sliding speed further increased, the COF of EP-P suddenly decreased to 0.16–0.17. This was due to the low S_mr1_ of EP-P, and led to frictional heat effects, causing thermal softening of EP. However, the bulk EP still maintained its integrity. The strip-like carbon transfer layer was developed on the counterpart surface parallel to the sliding direction (Figure 5b), caused by the initial wear. The thermal diffusivity of EP-P was 0.064 mm^2^/s. The higher thermal diffusivity correlates to effective frictional heat dissipation. Therefore, the specific wear rate of EP-P was maintained in the range from 6.0 × 10^−7^ to 6.6 × 10^−7^ cm^3^/Nm, indicating that it is unaffected by sliding speed.

For EP-F, the COF decreased from 0.25 to 0.16 with increasing sliding speed. The wear rate of EP-F at the sliding speed of 500 rpm is relatively low (5.5 × 10^−6^ cm^3^/Nm). This indicated uneasy wear under non-severe conditions from effective load-carrying, low thermal effects, and low sliding speed of EP. As the sliding speed increased to 1500 rpm, the surface temperature rose rapidly, and the wear behavior became severe. A large amount of wear debris of EP-F on the counterpart surface was generated, as shown in Figure 5d. When the debris powder scattered on the counterpart surface, it may have provided a three-body abrasion wear [36], which, in turn, contributed to a specific wear rate increase of EP-F (from 5.5 × 10^−6^ to 5.6 × 10^−5^ cm^3^/Nm). As shown in Table 4, the thermal absorptivity of EP-F was 371.9 Ws^1/2^/m^2^K, which may cause thermal softening of EP resin and a large amount of wear debris formation through abrasive wear. Due to the high wear loss generated at higher frictional heat, the catastrophic fracture of EP-F was likely to occur after a test period of 10,000 cycles under severe conditions at a sliding speed of 1500 rpm.

The staple carbon fiber fabric is a highly porous material. When it is partially-impregnated, the porous structure remains intact. This helps sustain structural integrity of the resin during wear process and reduced specific wear rate.

### 3.3. Effect of Applied Pressure on Friction Coefficient and Specific Wear Rate

**Figure 7:**
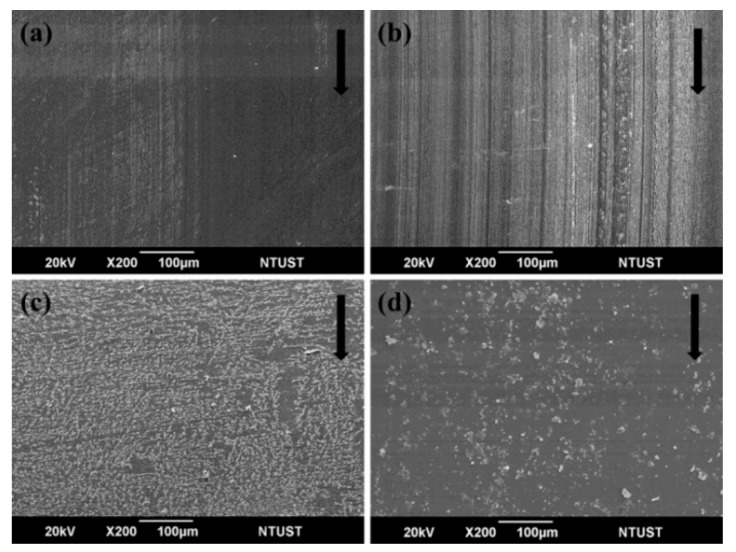
SEM images of the transfer phenomena on the counterpart surface of (**a**) PC-P, (**b**) EP-P, (**c**) PC-F, and (**d**) EP-F under a sliding speed of 1000 rpm and applied pressure of 1.5 MPa. The black arrows indicate the sliding direction.

The COF and specific wear rate of sCFC as a function of applied pressure under a constant sliding speed of 1000 rpm are summarized in Table 5. COFs of PC-based sCFC are higher than the one from EP-based sCFC, indicating that the polymer properties are the primary influence factor for COF. As the applied pressure increased, not only the COFs of sCFC slightly decreased (except for PC-F), but also appear to be insensitive to load. In this study, the highest COF is found to be 0.29 for PC-P. In general, the carbon fiber-reinforced composites have a COF in the range of 0.15–0.35 [26,27,37], which agrees with the COF obtained in this work. For the PC-P, COF decreased from 0.29 to 0.27 when the applied pressure increased. A few carbon-based transfer layers to the counterpart surface were observed (Figure 7a). PC-P had a lower specific wear rate, between 8.4 × 10^−7^ to 2.5 × 10^−6^ cm^3^/Nm. This is because carbon fibers are a solid lubricant, and discontinuous carbon fibers have remarkable load-carrying capabilities [10].

For PC-F, the friction force increased as the applied pressure increased from 0.5 to 1.5 MPa, leading to a high friction heat generated and consequent thermal softening of the polymer. Additionally, as the applied pressure increased, the COF of PC-F decreased from 0.27 to 0.19. Furthermore, as the applied pressure increased to 1.5 MPa, a large number of transfer films on the counterpart surface were observed (Figure 7c), accelerating the decrease in COF. The specific wear rate of PC-F increased from 1.3 × 10^−5^ to 1.6 × 10^−5^ cm^3^/Nm when increasing the applied pressure. The development of the polymer transfer film was impacted by thermal softening that occurred at the sliding interface, caused by high thermal absorptivity characteristics of the PC-F. Also, the transfer film was caused either by the plastic deformation of PC resin or by adhesion of the polymer to the steel counterpart during sliding.

For EP-P, the COF decreased from 0.19 to 0.16 when increasing the applied pressure. As shown in Figure 7b, carbon-based transfer films and EP resin debris were present on the counterpart surface from the 1.5 MPa of applied pressure. The specific wear rate of EP-P slightly increased from 2.1 × 10^−7^ to 2.7 × 10^−6^ cm^3^/Nm, indicating that it was slightly impacted by the applied pressure.

For EP-F, the COF decreased from 0.21 to 0.19 with increasing applied pressure due to the friction force provided from debris generated by the three-body abrasive wear. In addition, as shown in Table 4, the higher thermal absorptivity (371.9 Ws^1/2^/m^2^K) and lower thermal diffusivity (0.036 mm^2^/s) of EP-F also caused both a thermal softening of EP resin and a large amount of wear debris. At an applied pressure of 0.5 MPa, the good load-carrying capability of EP and low thermal effect at low sliding speed caused the specific wear rate to become around 1.8 × 10^−6^ cm^3^/Nm. When the applied pressure was increased to severe conditions, the specific wear rate of EP-F increased from 1.8 × 10^−6^ to 3.2 × 10^−5^ cm^3^/Nm. A large amount of wear debris of EP-F on the counterpart surface was observed, as shown in Figure 7d.

In summary, as the sliding speed and/or applied pressure increases, the COF of the sCFC decreases, whereas the specific wear rate increases. These results are also found in the literatures [28,29,30]. However, it can be further improved with use of additives into the matrix or carbon fiber surface modification [15,23].

### 3.4. SEM Analysis of the Worn Surface

#### 3.4.1. The Influence of Partially-Impregnated Surface Topography on the Wear Mechanism

**Figure 8:**
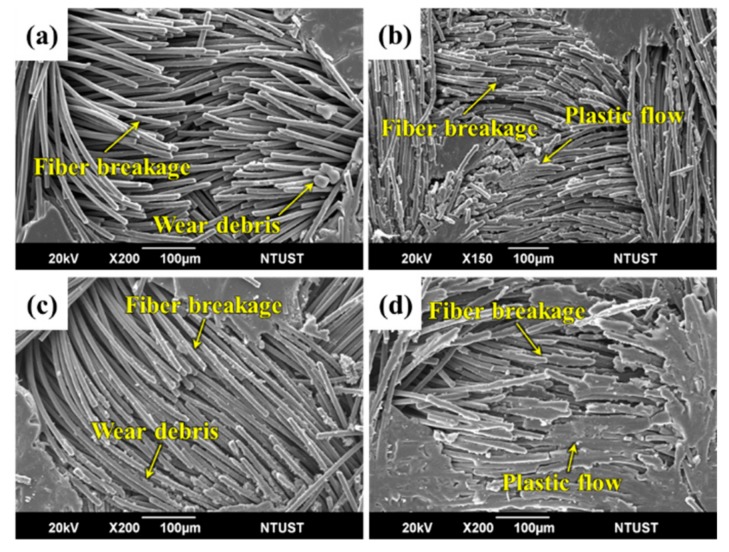
SEM images of the worn surface of PC-P at sliding speeds of (**a**) 500 rpm, (**b**) 1500 rpm, and applied pressures of (**c**) 0.5 MPa and (**d**) 1.5 MPa, respectively.

**Figure 9:**
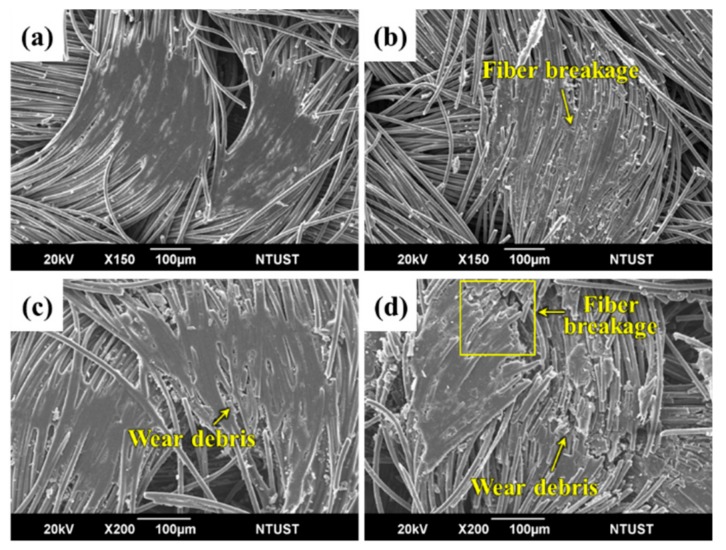
SEM images of the worn surface of EP-P at sliding speeds of (**a**) 500 rpm, (**b**) 1500 rpm, and applied pressures of (**c**) 0.5 MPa and (**d**) 1.5 MPa, respectively.

To study wear mechanisms, the worn surfaces of sCFCs with different surface topographical characteristics were observed in Figure 8 and Figure 9. For PC-P at 500 rpm, both a large number of fiber breakages and fewer wear debris occur in the protuberance zone of worn surface (Figure 8a). However, this sample kept its fiber bundle state as the surface continued to be rubbed. When the sliding speed increased to 1500 rpm, the contact temperature and wear loss also increased. This resulted in plastic flow from the PC-rich region and caused the carbon fibers to be coated with a large amount of resin. The broken fibers were accumulated between the warp and weft of fibrous bundles (Figure 8b).

For PC-P, with an applied pressure of 0.5 MPa, a similar wear mechanism was observed at low sliding speeds (Figure 8c). The friction contact area increased as the applied pressure further increased to 1.5 MPa. Additionally, the contact temperature also increased, which resulted in a plastic flow from the PC-rich region, causing the carbon fibers to be coated with resin (Figure 8d).

For EP-P, a small amount of wear debris accumulated in the valley of the staple carbon fiber fabric at 500 rpm. Fiber breakages occurred in the non-impregnated carbon fiber, whereas good interface bonding was found in carbon fiber bundles impregnated with EP resin (Figure 9a). When the sliding speed increased to 1500 rpm, fiber breakages and wear debris were observed (Figure 9b).

At an applied pressure of 0.5 MPa, few fiber breakages and fiber/matrix debonding were found (Figure 9c). When the applied pressure increased to 1.5 MPa, a large number of fiber breakages and wear debris were observed on the worn surface (Figure 9d). Carbon fiber-dominated surface topography helped sustain the structural integrity during friction and wear process, thereby reducing the specific wear rate. 

#### 3.4.2. The Influence of Fully-Impregnated Surface Topography on Wear Mechanism

**Figure 10:**
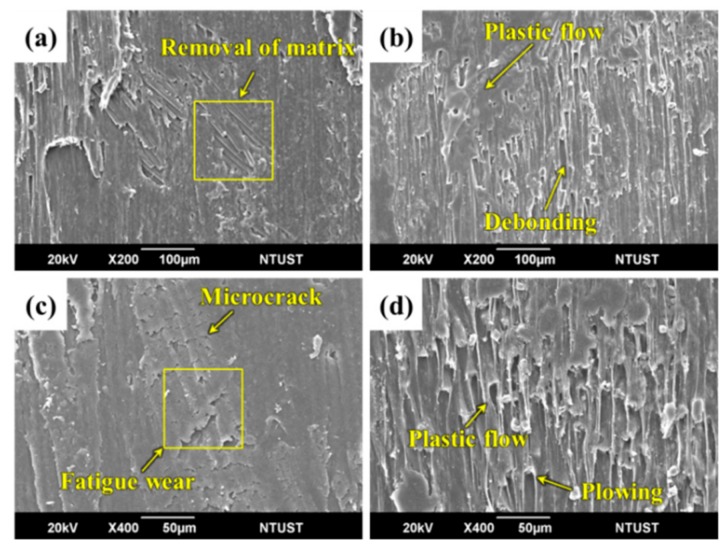
SEM images of worn surface of PC-F at sliding speeds of (**a**) 500 rpm, (**b**) 1500 rpm, and applied pressures of (**c**) 0.5 MPa and (**d**) 1.5 MPa, respectively.

Different wear mechanisms were observed in different surface topographical characteristics of carbon fabric composites. For PC-F at 500 rpm, removal of PC resin from the surface and fiber/matrix interface debonding was found (Figure 10a). When the sliding speed increased to 1500 rpm, greater fiber/matrix interface debonding occurred, resulting in wear debris formation and fiber breakages. This was due to the weak adhesive present between carbon fibers and PC resin. The high contact temperature softened the resin, inducing the plastic flow deformation of PC resin in the process (Figure 10b).

For PC-F at an applied pressure of 0.5 MPa, resin abrasion wear and extensive formation of surface microcracks can be observed. As the number of engagement cycles increased, microcracks propagated, forming a network of interconnected cracks under continuously applied pressure, and leading to surface fatigue wear (Figure 10c). After a number of cycles, fatigue wear dominates the wear behavior and results in severe material loss. This mechanism appears to be more important when higher loads are employed [38]. When the sliding speed increased to 1.5 MPa, the damage to the fiber and PC resin was severe. Serious plastic flow deformation occurred in the sliding direction, which led to frictional heat developing during the sliding process [39], thus increasing to eventual fiber breakages. The groove was caused by the micro-plowing action of removing broken carbon fibers (Figure 10d). Therefore, the main wear mechanisms of PC-based composites are abrasive and adhesive wear.

**Figure 11:**
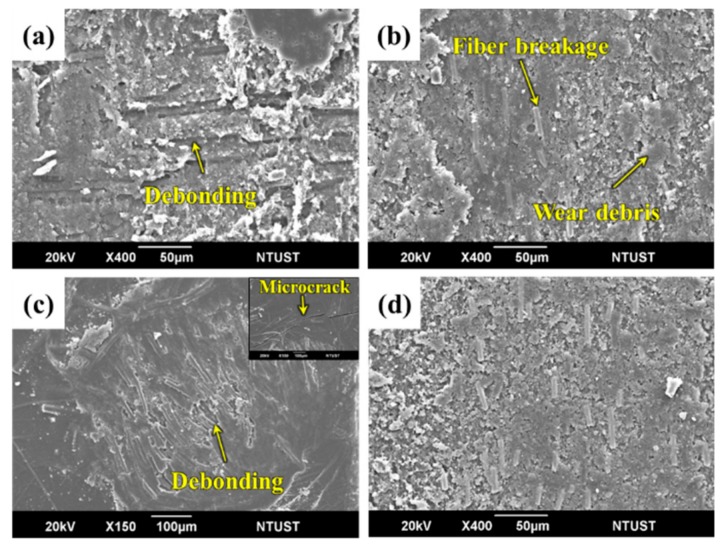
SEM images of worn surface of EP-F at sliding speeds of (**a**) 500 rpm, (**b**) 1500 rpm, and applied pressures of (**c**) 0.5 MPa and (**d**) 1.5 MPa, respectively.

For EP-F at 500 rpm, the thermoset resin structure of the EP resin was unable to form a plastic flow like the one observed in PC resin. The carbon fabric composites underwent a micro-plowing action of the counterpart surface to generate a large amount of wear debris. This caused a three-body frictional behavior. As resin is removed, the carbon fiber undergoes a micro-cutting action of the wear debris. This results in fiber breakage and the fiber/matrix interface debonding to form grooves (Figure 11a). When the sliding speed increased to 1500 rpm, frictional heat raised rapidly, causing severe fiber breakage and wear debris. This also caused high wear loss due to three-body frictional behavior (Figure 11b).

**Figure 12:**
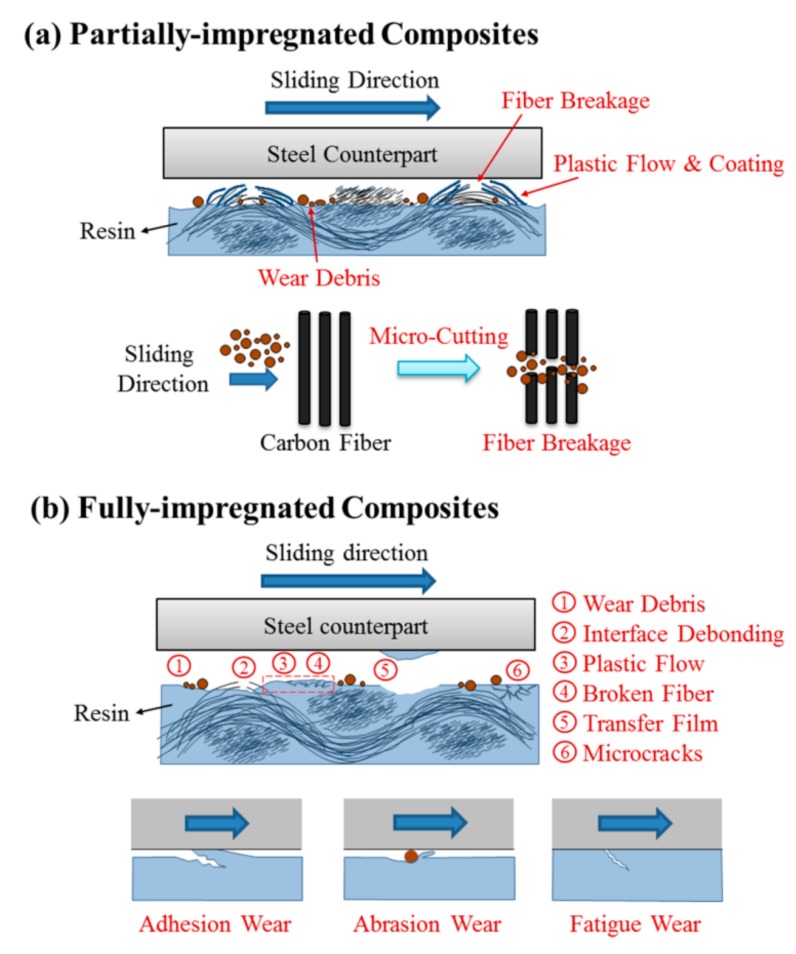
Schematic wear mechanisms of the (**a**) partially-impregnated and (**b**) fully-impregnated stable carbon fabric composites (sCFC).

For EP-F at an applied pressure of 0.5 MPa, fibers were well embedded into the matrix. Additionally, the repeated mechanical and thermal stresses induced fiber/matrix debonding and fiber breakage of the protuberance zone. It was noticed that a typical fatigue wear mechanism occurred in this process, which induced matrix cracks on the surface (Figure 11c). When the applied pressure increased to 1.5 MPa, the complex wear mechanisms participated in the wear process and a large amount of wear debris was found on the worn surface (Figure 11d). Therefore, the main wear mechanism of EP-based composites was concluded to be abrasive wear. Figure 12 illustrates the schematic wear mechanisms of the partially-impregnated and fully-impregnated composites.

## 4. Conclusions

In this study, the influence of resin type and resin impregnated level on tribological properties and wear mechanisms for staple carbon fabric composites was investigated. The major outcomes are outlined as follows:

- The tribological tests revealed that the resin type acts as the primary influence factor for friction coefficient, followed by the S_mr1_ and the S_k_ values. The friction coefficient of composites decreased with increasing the sliding speed/applied pressure and depended on surface topography and temperature at the worn surface. Additionally, the partially-impregnated composites present a steady-state friction coefficient curve, while the fully-impregnated composites present a large fluctuation on the amplitude of the friction coefficient during the friction process.
- On the other hand, the primary influence factor for the specific wear rate was observed to be the level of resin impregnation. The partially-impregnated composites maintained a lower specific wear rate when increasing the sliding speed/applied pressure. However, the specific wear rate of fully-impregnated composites substantially increased with increasing sliding speeds and/or applied pressures. Carbon fabric composites under severe friction conditions, such as 1500 rpm or 1.5 MPa, largely increased their respective specific wear rate.
- Finally, for PC-based composites, the main wear mechanisms were determined to be abrasive, adhesive, and fatigue wears. Conditions for each wear mechanism include matrix wear, plastic flow, towing fracture of resin, squeezing deformation of resin, plowing fracture, transfer film, fiber/matrix debonding, and fiber breakage. For EP-based composites, the main wear mechanisms included abrasive wear and fatigue wear. Conditions for each wear mechanism include micro-cutting fracture, plowing fracture, fiber/matrix debonding, fiber breakage, and crushing fracture.

## Figures and Tables

**Table 1:** Composition friction (vol%) of hybrid composites.

Sample Codes	Carbon Fiber (vol%)	Glass Fiber (vol%)	PC (vol%)	Epoxy (vol%)	Porosity (vol%)
PC-P	29	5	27	-	39 ± 2
PC-F	28	5	67	-	0
EP-P	28	5	-	21	46 ± 2
EP-F	27	5	-	68	0

**Table 2:** Surface roughness parameters for polycarbonate (PC)- and EP-based composites.

Sample	S_q_ (μm)	S_k_ (μm)	S_pk_ (μm)	S_mr1_ (%)
PC-P	23.95 ± 1.57	49.28 ± 6.73	22.32 ± 2.41	14.10 ± 0.51
PC-F	4.39 ± 0.30	10.22 ± 0.68	5.37 ± 0.50	11.70 ± 0.58
EP-P	51.25 ± 2.66	69.40 ± 9.28	32.43 ± 2.65	7.36 ± 0.67
EP-F	4.23 ± 0.18	9.98 ± 0.59	5.54 ± 0.30	13.25 ± 0.70

**Table 3:** Friction coefficients and specific wear rates for each of the four materials with different sliding speed.

Sample	Coefficient of Friction (COF)			Specific Wear Rate (10^−7^ cm^3^/Nm)		
	500 rpm	1000 rpm	1500 rpm	500 rpm	1000 rpm	1500 rpm
PC-P	0.28 ± 0.03	0.27 ± 0.02	0.21 ± 0.01	11.0 ± 4.0	8.4 ± 4.3	15.3 ± 3.7
PC-F	0.26 ± 0.04	0.23 ± 0.02	0.14 ± 0.03	92.9 ± 8.2	152.0 ± 17.7	209.0 ± 43.4
EP-P	0.24 ± 0.01	0.17 ± 0.01	0.16 ± 0.01	6.0 ± 2.2	6.6 ± 1.5	6.2 ± 3.5
EP-F	0.25 ± 0.03	0.20 ± 0.02	0.16 ± 0.02	55.0 ± 7.9	242.0 ± 29.2	563.0 ± 64.0

**Table 4:** Thermal properties for each of the four materials.

Sample	Thermal Diffusivity (10^−3^ mm^2^/s)	Thermal Absorptivity (Ws^1/2^/m^2^K)
PC-P	53 ± 4	314.7 ± 10.3
PC-F	46 ± 4	404.7 ± 14.8
EP-P	64 ± 7	236.7 ± 11.9
EP-F	36 ± 1	371.9 ± 11.5

**Table 5:** The friction coefficient and specific wear rate for each of the four materials with different applied pressure.

Sample	Coefficient of Friction (COF)			Specific Wear Rate (10^−7^ cm^3^/Nm)		
	0.5 MPa	1.0 MPa	1.5 MPa	0.5 MPa	1.0 MPa	1.5 MPa
PC-P	0.29 ± 0.01	0.27 ± 0.02	0.27 ± 0.02	24.8 ± 5.3	8.4 ± 4.3	16.3 ± 5.3
PC-F	0.27 ± 0.02	0.23 ± 0.02	0.19 ± 0.02	129.0 ± 9.8	152.0 ± 17.7	164.0 ± 27.2
EP-P	0.19 ± 0.02	0.17 ± 0.01	0.16 ± 0.01	2.1 ± 0.3	6.6 ± 1.5	26.6 ± 6.5
EP-F	0.21 ± 0.02	0.20 ± 0.02	0.19 ± 0.04	17.8 ± 5.0	242.0 ± 29.2	317.0 ± 25.2
